# The Effect of Thickness of Resorbable Bacterial Cellulose Membrane on Guided Bone Regeneration

**DOI:** 10.3390/ma10030320

**Published:** 2017-03-21

**Authors:** You-Jin Lee, Sung-Jun An, Eun-Bin Bae, Hui-Jeong Gwon, Jong-Seok Park, Sung In Jeong, Young-Chan Jeon, So-Hyoun Lee, Youn-Mook Lim, Jung-Bo Huh

**Affiliations:** 1 Department of Prosthodontics, Dental Research Institute, Institute of Translational Dental Sciences, BK21 PLUS, School of Dentistry, Pusan National University, Yangsan 50612, Korea; nicejin17@naver.com (Y.-J.L.); 0228dmqls@hanmail.net (E.-B.B.); jeonycdds@hanmail.net (Y.-C.J.); romilove7@hanmail.net (S.-H.L.); 2 Advanced Radiation Technology Institute, Korea Atomic Energy Research Institute, 1266 Sinjeong-dong, Jeongeup-si, Jeollabuk-do 56212, Korea; asj@kaeri.re.kr (S.-J.A.); hjgwon@kaeri.re.kr (H.-J.G.); jspark75@kaeri.re.kr (J.-S.P.); joung5295@hanmail.net (S.-I.J.); ymlim71@kaeri.re.kr (Y.-M.L.)

**Keywords:** bacterial cellulose, guided bone regeneration, membrane, resorbable membrane, thickness

## Abstract

This study introduces the effect of the thickness of a bacterial cellulose membrane by comparing the bone regeneration effect on rat skulls when using a collagen membrane and different thicknesses of resorbable bacterial cellulose membranes for guided bone regeneration. Barrier membranes of 0.10 mm, 0.15 mm, and 0.20 mm in thickness were made using bacterial cellulose produced as microbial fermentation metabolites. Mechanical strength was investigated, and new bone formation was evaluated through animal experimental studies. Experimental animals were sacrificed after having 2 weeks and 8 weeks of recovery, and specimens were processed for histologic and histomorphometric analyses measuring the area of bone regeneration (%) using an image analysis program. In 2 weeks, bone-like materials and fibrous connective tissues were observed in histologic analysis. In 8 weeks, all experimental groups showed the arrangement of osteoblasts surrounding the supporting body on the margin and center of the bone defect region. However, the amount of new bone formation was significantly higher (*p* < 0.05) in bacterial cellulose membrane with 0.10 mm in thickness compared to the other experimental groups. Within the limitations of this study, a bacterial cellulose membrane with 0.10 mm thickness induced the most effective bone regeneration.

## 1. Introduction

Guided bone regeneration (GBR) is a conventional procedure that places a barrier membrane over a bone defect either filled with or without bone graft materials [1,2,3]. An enclosed space is created by the barrier membrane, allowing only osteogenic cells to populate the bone defect [4,5]. Then, the barrier membrane excludes surrounding tissues that derive unwanted re-growth of the gingival epithelium and connective tissue cells [5,6].

Barrier membranes are required to possess properties such as biocompatibility, cell-occlusiveness, space maintenance ability, tissue integration, and manageability [7]. Numerous barrier membranes have been introduced, and barrier membranes can be classified as resorbable or non-resorbable membranes [8,9,10]. Although non-resorbable barrier membranes draw a predictable result because of their space maintenance ability, they manifest some disadvantages, including a second surgery necessary for removal and risk of infection due to external exposure. Expanded-polytetrafluoroethylene (e-PTFE) and titanium mesh are widely used as non-resorbable membranes [11,12,13,14]. As a result, resorbable barrier membranes are often used in clinical trials, and collagen membranes are recently used as biodegradable barriers in GBR [15,16]. Since collagen membranes do not require a second surgery and display excellent hemostasis, early wound stabilization, chemotaxis for fibroblasts, and suitable tension, they are widely used [17]. Nonetheless, they possess limitations, such as lack of space-making ability, unpredictable biodegradation period, and high cost. To date, many studies on GBR membranes have been carried out in various fields [18].

Cellulose is a polysaccharide macromolecule with β-(1,4) glycosidic bonds, and it is a source of cytoderms in higher plants [19]. Heteropolysaccharides in plant cellulose become attached with other polysaccharides that ultimately decrease mechanical strength and absorbability [20,21]. Cellulose can be produced by bacteria, and bacterial cellulose (BC)—which is produced by the internal bacteria like *Agrobacterium*, *Pseudomonas*, *Rhizobium*, and *Komagataeibacter*—does not contain hemicellulose, pectin, lignin, or biogenic products. Compared to plant cellulose, BC exhibits physicochemical properties including high purity, high crystallinity, high mechanical strength, high hydrophilicity, excellent biocompatibility, and outstanding biodegradability [22,23]. Incorporating these advantageous properties of BC, new materials have been developed in many industries, including pharmaceutical companies, and one of them was the development of new barrier membrane for GBR [22,23,24]. A previous study comparing collagen membranes to BC membranes suggested that the BC membrane can take the role of a barrier membrane [25]. BC membranes demonstrate properties such as swelling with high hydrophilicity and exchange of oxygen and nutrients through the micropores composed of microfibers [26]. BC membranes can be synthesized in greater thickness under extended incubation time. Depending on the thickness of the membrane, it is possible to control the permeability for sufficient fluid and gas [27,28,29].

Therefore, in this study, the mechanical properties of BC membranes of various thicknesses were measured, and a GBR procedure was then performed in rat calvarial defect models in order to investigate the proper thickness of BC membranes for GBR. 

## 2. Results

### 2.1. Thickness Measurement

BC membranes were reprocessed in film form by freeze-drying and irradiating. The dried BC membranes were then cut into fixed size of 20 mm in length and 15 mm in width (n = 5). Errors in the length and width of the BC membranes were less than 0.10 mm. However, the thicknesses of BC membranes were fabricated differently for each sample: they were designed in 0.10 mm, 0.15 mm, and 0.20 mm thicknesses. The thickness of the collagen membrane was measured as 0.317 ± 0.026 mm, while the thicknesses of the BC membranes were measured as 0.103 ± 0.017 mm, 0.151 ± 0.012 mm, and 0.207 ± 0.006 mm. This might be caused by differences in BC culture conditions, such as incubation time, medium conditions, and culture temperature.

### 2.2. Scanning Electron Microscopy (SEM)

**Figure 1:**
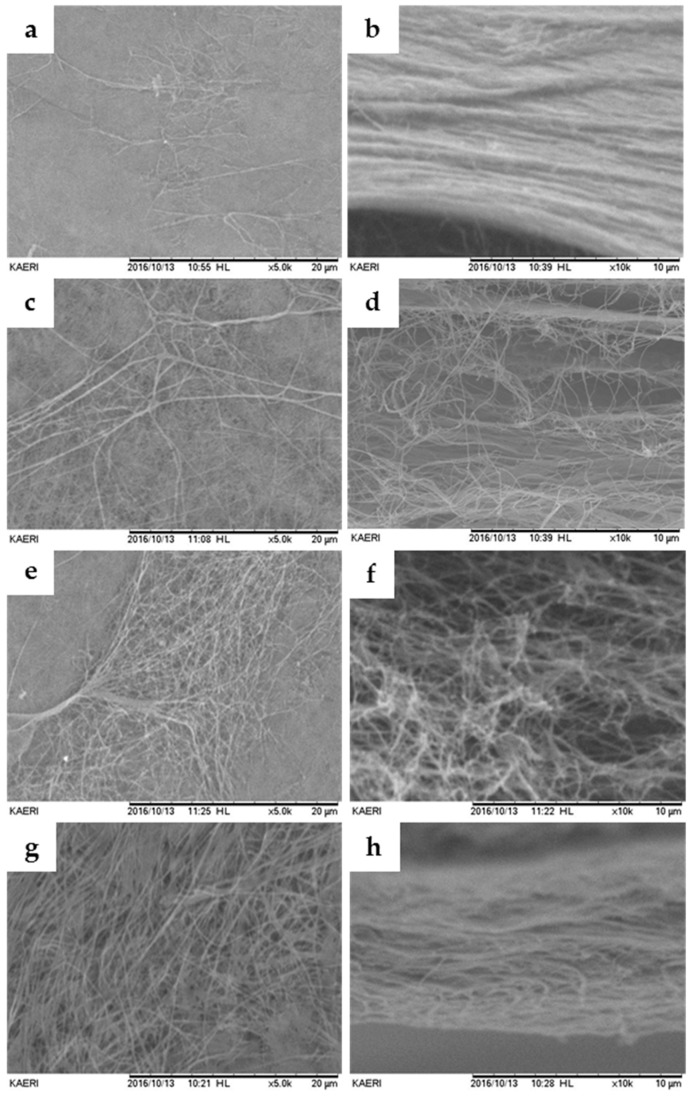
Scanning electron microscope (SEM) images of the surface and cross-section of bacterial cellulose (BC) and collagen membranes. (**a**,**b**) Collagen membrane; (**c**,**d**) 0.10 mm BC membrane; (**e**,**f**) 0.15 mm BC membrane and (**g**,**h**) 0.20 mm BC membrane (original magnification: surface, ×5k (a,c,e,g); cross-section, ×10k (b,d,f,h)).

The results from SEM analysis showed that the microstructures of the BC and collagen membranes were a network structure composed of micro-sized fibers, as shown in Figure 1. In addition, the structures of the surface and cross-section of BC membranes were similar to those of the collagen membrane. Therefore, more fibers are exposed to the surface of the BC membrane as the BC membrane gets thicker. 

### 2.3. Mechanical Test of Membranes

**Figure 2:**
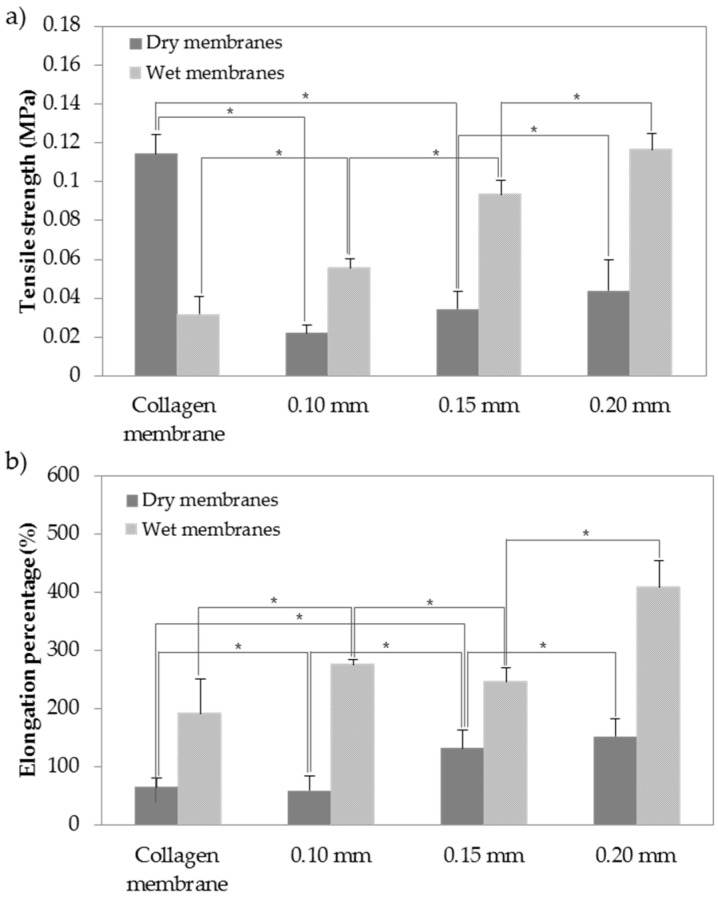
Mechanical properties of BC and Collagen membranes: (**a**) Tensile strength (MPa) of dry and wet membranes; (**b**) Elongation percentage (%) of dry and wet membranes. The “*” symbol indicates a significant difference (*p* < 0.05).

The mechanical properties of artificial matrix in tissue engineering are critical due to the necessity of structural stability to withstand stress incurred during implanting in vivo. Such properties as tensile strength and elongation percentage can also significantly affect some specific biological functions of cells in implanted tissue [30]. Therefore, tensile strengths and elongations of BC and collagen membranes were observed as shown in Figure 2. In general, mechanical strengths of dried membranes were higher than those of wet membranes. According to the experimental measurements of tensile strength and elongation, tensile strengths of the wet membranes were higher than those of the dried membranes. In particular, there was a significant difference between elongation of the collagen membrane and elongation of BC membranes (collagen: 380% ± 6%, 0.10 mm: 275% ± 7.5%, 0.15 mm: 246% ± 3%, 0.20 mm: 408% ± 7%) (*p* < 0.05). Elongation of BC membranes was significantly greater compared to that of collagen membrane. In addition, BC membranes showed greater strength properties, even though they were thinner than collagen membrane. These characteristics may be due to the hydrogen bonds between the fibrils of BC [31].

### 2.4. Histological Findings

**Figure 3:**
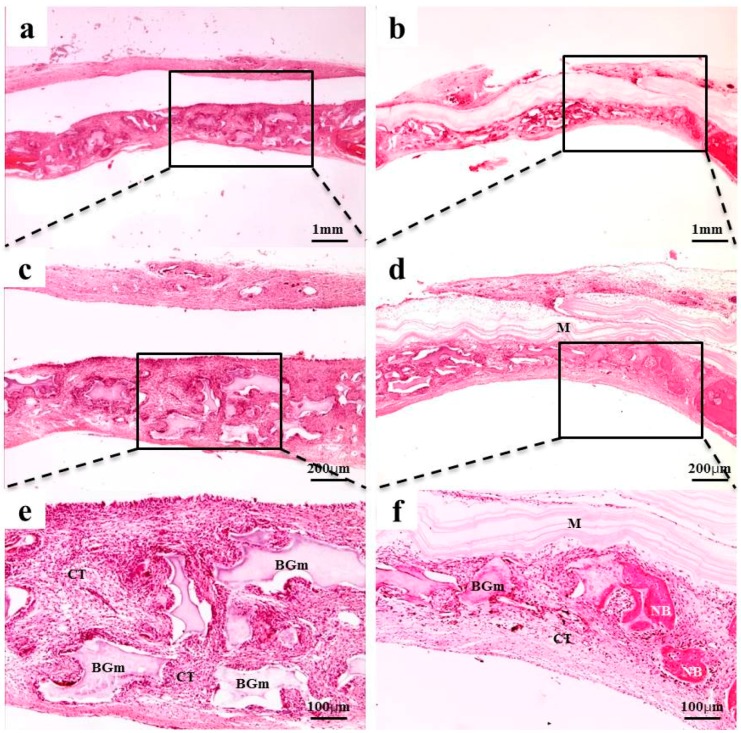
Collagen and 0.10 mm BC membrane. Histological sections of defect sites at 2 weeks after surgery: (**a**,**c**,**e**) collagen membrane; (**b**,**d**,**f**) 0.10 mm BC. Fibrous connective tissues and bone-like materials were observed in all groups. NB, Fibrous connective tissue; M, membrane; CT, connective tissue; BGm, bone grafting material. Original magnification: ×20 (a,b), ×40 (c,d), ×100 (e,f).

**Figure 4:**
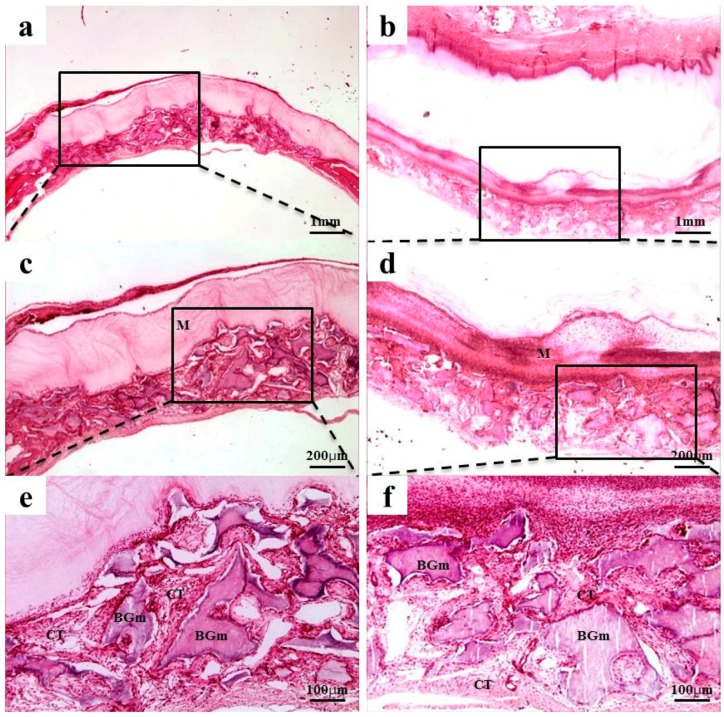
BC membranes: 0.15 mm and 0.20 mm. Histological sections of defect sites at 2 weeks after surgery: (**a**,**c**,**e**) 0.15 mm BC; (**b**,**d**,**f**) 0.20 mm BC. Fibrous connective tissues and bone-like materials were observed in the 0.15 mm BC group. NB, Fibrous connective tissue*;* *M,* membrane; CT*,* connective tissue; BGm, bone grafting material. Original magnification: ×20 (a,b), ×40 (c,d), ×100 (e,f).

**Figure 5:**
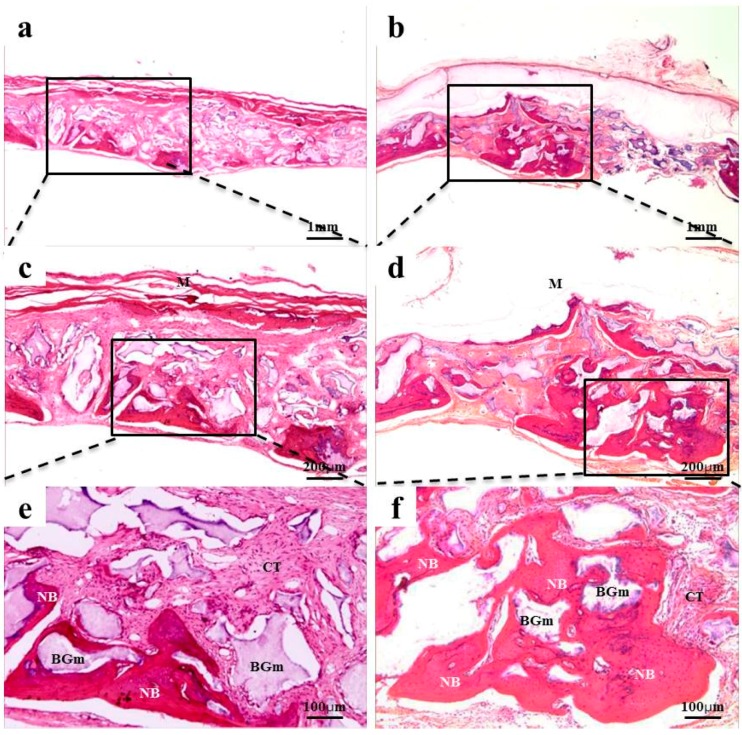
Collagen membrane and 0.10 mm BC membrane. Histological sections of defect sites at 8 weeks after surgery: (**a**,**c**,**e**) collagen membrane; (**b**,**d**,**f**) 0.10 mm BC. New bone formations were observed in all groups. NB, Fibrous connective tissue; M, membrane; CT, connective tissue; BGm, bone grafting material. Original magnification: ×20 (a,b), ×40 (c,d), ×100 (e,f).

**Figure 6:**
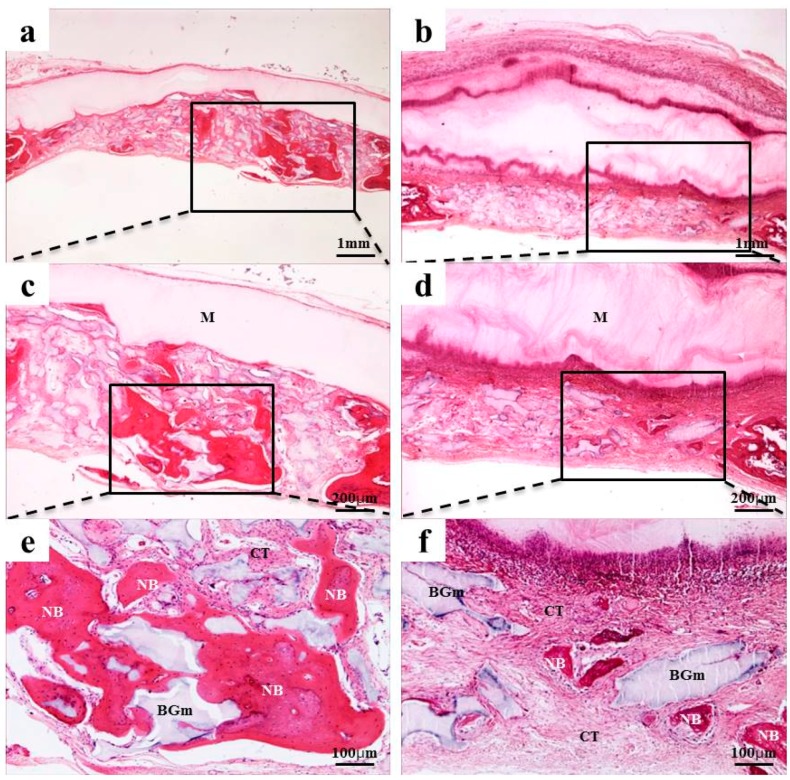
BC membranes: 0.15 mm and 0.20 mm. Histological sections of defect sites at 8 weeks after surgery: (**a**,**c**,**e**) 0.15 mm BC; (**b**,**d**,**f**) 0.20 mm BC. NB, Fibrous connective tissue*;* *M,* membrane; CT*,* connective tissue*;* BGm*,* bone grafting material. Original magnification: ×20 (a,b), ×40 (c,d), ×100 (e,f).

**Figure 7:**
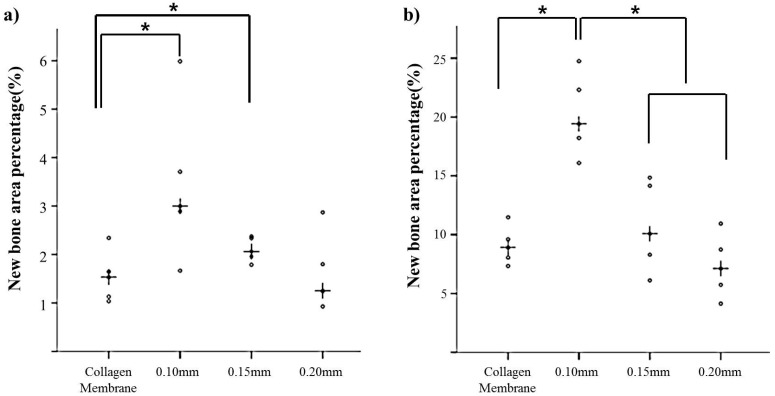
Scatter plot and median (the cross) representing (**a**) 2 weeks after surgery new bone area percentage; (**b**) 8 weeks after surgery new bone area percentage. The symbol “*” indicates significantly different (*p* < 0.05).

After 2 weeks of recovery, the defect region was isolated by a membrane, and generations of fibrous connective tissue and bone-like material were observed through microscope. In the 0.10 mm BC membrane group, the new bone formation was observed in the margins of old bone boundaries and around bone graft materials, but the newly-formed bone was immature (Figure 3b,d,f). In the 0.20 mm BC membrane group, membrane expansion and plasma exudation in the upper structure were observed, but new bone formation was not observed in the lower membrane structure (Figure 4b,d,f). In all experimental groups sacrificed at 8 weeks after surgery, the new bone formation was generated greatly and also observed in the peripherals of bone graft materials. Mesenchymal cells, fibrous connective tissues, and osteoblasts were observed in the peripherals of the new bone substrate (Figure 5 and Figure 6). Comparing the groups with different materials, post-hoc analysis showed a significant difference between collagen and 0.10 mm BC groups (*p* < 0.01) at 2 weeks after surgery. There was a significant difference between collagen and 0.15 mm BC groups. A difference among the remaining groups was not significant (collagen: 1.54% ± 0.52%, 0.10 mm: 3.45% ± 1.60%, 0.15 mm: 2.10% ± 0.25%, 0.20 mm: 1.62% ± 0.77%) (*p* > 0.05). After 8 weeks of healing, the mean value of new bone formation in the 0.10 mm BC group (Figure 5b,d,f) was particularly significant among the three groups, while there was no significant difference among the collagen group, 0.15 mm BC group, and 0.20 mm BC group (collagen: 9.08% ± 1.59%, 0.10 mm: 20.16% ± 3.41%, 0.15 mm: 10.70% ± 3.75%, 0.20 mm: 7.33% ± 2.63%) (*p* < 0.05, Figure 7). After 2 weeks and 8 weeks of healing, the average neo-tissue (NT)/ new-bone (NB) area (%) of sacrificial tissue was measured as presented in Table 1.

## 3. Discussion

Barrier membranes can be classified into resorbable and non-resorbable membranes depending on their absorption characteristics [8,9,10]. Non-resorbable membranes provide predictable results due to their excellent space-maintenance ability. However, since they are not resorbed in vivo, they should be removed 4 to 6 weeks after surgery through a second surgery [32,33]. An immature regenerated bone below a barrier membrane proliferates and attaches to the membrane. Therefore, a second surgery inevitably causes mechanical damage on the regenerated bone during a healing period. In addition, when the regenerated bone cannot be completely covered with a flap after the second surgery, the regenerated bone can be also decreased [32,33]. Herein, resorbable barrier membranes that do not require a second surgery have interested many clinicians and motivated researchers to execute extended research with the purpose of resolving the problem of non-resorbable membranes. Resorbable barrier membranes have brought benefits in reducing burdens on both patients and surgeons [11,12].

Ideal resorbable barrier membranes should not be removed by a second surgery in order to maintain their state, even after completing tissue regeneration. They should also block tissue penetration effectively and not cause tissue rejection and allergy while displaying infection resistance and manageability [34]. Dahlin et al. [5] suggested that resorbable barrier membranes may cause a local inflammatory response involving phagocytosis and that cell occlusion and space maintenance abilities are inferior to those of non-resorbable barrier membranes. Resorbable membranes may be absorbed early before completing periodontal regeneration.

Minabe et al. [35] found that the first 3 to 4 weeks of recovery after surgery are critical for tissue regeneration, and that a barrier membrane should not be absorbed too quickly. The study also reported that inflammatory response during the absorption process should not interfere with bone formation or maturation. In addition, the study left no definite conclusion about the optimal timings of barrier membrane absorption and the degradation of barrier membrane structure. Furthermore, the molecular weight, composition, surface area, porosity, and cross-linking technique of the barrier membrane were used to control the absorption rate of the barrier membrane [35].

Currently, the most commonly used collagen membranes (CM) show good biocompatibility, and they retain great advantages including excellent manageability and no requirement for a second surgery [15,16,17]. However, CMs have the disadvantage of high cost, so many studies have been done to replace CMs in various fields; one of them is BC membrane, which is widely found in nature [36]. BC is a water-insoluble extracellular polysaccharide with a simple structure [19]. The β-(1,4) glucan chains form microfibrils, which are crystallized into cellulose fibers [20]. Compared to plant cellulose, BC demonstrates superior physicochemical properties; e.g., high purity, high crystallinity, high mechanical strength, high hydrophilicity, and good biocompatibility and biodegradability. In addition, the cost of producing BC membrane is substantially low, and it also shows high-purity isolation [21,22,23]. In a previous study, Helenius et al. [37] conducted an experiment on the biocompatibility of BC, and Lee et al. [27] investigated the role of BC membrane as a barrier membrane in GBR, comparing it to collagen membrane. Since BC membrane was developed, various types of BC membranes have been produced through culture, irradiation, introduction of gelatin into nano-fibers, and freeze-drying or hot air drying. In addition, many attempts have been made to find the optimal form for GBR [36]. BC membranes have properties such as swelling due to high hydrophilicity and exchange of oxygen and nutrients through the micropores composed of microfibers [26]. BC membranes can be synthesized thicker with a longer incubation time [27]. Depending on the thickness of the membrane, it is possible to control the permeability for adequate fluid and gas exchange [27,28,29]. Therefore, the purpose of this study was to investigate the appropriate thickness.

BC membranes of different thickness were irradiated to make resorbable membranes, and then a mechanical experiment was performed. SEM analysis showed that the thicker the membrane, the greater the amount of fiber. However, BC membranes and collagen membranes were similar in cross-section images. In the tensile strength test, the dry tensile strength of the BC membrane was smaller than that of collagen, but the wet tensile strength was higher than that of collagen. It had sufficient operability for clinical use. In addition, the elongation percentage of BC membrane was larger than that of collagen, although it was thinner than the collagen membrane. This may be due to the hydrogen bonds of BC membrane fibrils [38].

At 2 weeks of in vivo experiment, plasma exudate and inflammatory cells were observed in the 0.20 mm BC membrane group, and new bone formation was not observed in the lower part of the membrane. Among the 8 week sacrificed tissues, the new bone formation percentage was significantly higher in the 0.10 mm BC membrane group than in the other three groups (*p* < 0.001). In addition, there was no significant difference among the collagen membrane group, 0.15 mm, and 0.20 mm BC membrane groups. Previous studies suggested that the barrier membrane used in GBR should be permeable enough to allow transfer of nutrients and air. Hurley et al. [39] reported that in the animal study involving vertebrae fusion, the site with impermeable rubber silicon membrane was not healed by bone formation, whereas the site with microporous cellulosic acetate membrane was completely recovered through bone formation. The cellulose acetate membrane could transfer tissue fluid into bone marrow, feeding nutrients through the membrane and providing a better environment for bone remodeling. Membrane permeability was decreased as swelling was increased in the 0.20 mm BC membrane group. The 0.10 mm BC membrane group showed the highest level of new bone volume (mm³) and new bone area percentage (%).

Histological sections of the 0.20 mm BC membrane group exhibited swelling of the membrane, which may be responsible for decreased nutritional supply, fluid permeability, and bone regeneration ability. Moreover, the 0.10 mm BC membrane group demonstrated the greatest permeability, while it shielded the defect area successfully without getting absorbed until 8 weeks after surgery, ultimately manifesting the greatest level of new bone volume (mm³) and new bone area percentage (%). Along with biomolecule transport and revascularization, cell adhesion and permeability play crucial roles in new bone formation. Therefore, further studies are required to study cell proliferation and the mechanical properties of the membranes.

## 4. Materials and Methods

### 4.1. Preparation of Bacterial Cellulose Membrane

The initial thicknesses of the BC membranes were 5 mm, 7 mm, and 9 mm, respectively. BC membranes were provided from Jadam Co. (Jeju, Korea). The bacterial strain *Komagataeibacter hansenii* TL-2C was incubated for 7, 9, and 12 days in a static culture containing 0.3% (*w*/*w*) citrus fermented solution and 5% (*w*/*w*) sucrose. The pH was adjusted to 4.5 with acetic acid. The obtained BC pellicles were purified by immersion in deionized water at 90 °C for 2 h and then boiled in a 0.5 M aqueous solution of NaOH for 20 min to remove bacterial cell remains. The BCs were then washed with deionized water several times and soaked in 1% NaOH for 2 days. Finally, the BC pellicles were washed free of alkali. All the other reagents and solvents were of analytical grade and used without further purification. The BCs in distilled water were irradiated in room temperature with an electron beam linear accelerator (10 MeV, 0.5 mA) at the Korea Atomic Energy Research Institute (Jeongup, Korea) for preparation of BC resorbable membrane, at irradiation doses up to 100 kGy with a dose rate of 5 kGy/min. After the irradiation, the BCs were washed with deionized water and then freeze-dried at −80 °C for 48 h after being fixed between the stainless steel wire meshes in order to remove water.

### 4.2. Thickness Measurement

In this study, the thicknesses of BC membranes were measured with vernier calipers (Mitutoyo Co., Kawasaki, Japan) and thickness gauge (Mitutoyo Co., Kawasaki, Japan), respectively. Collagen membrane (Ossix plus, Lod, Islael) was measured as the control. The thickness of each membrane was tested at three different points by the same observer (n = 5). The results were expressed as the mean values of three determinations of the BC membrane.

### 4.3. Mechanical Properties

BC membranes and collagen membrane (Ossix plus, Lod, Islael) were evaluated for their mechanical properties using a Universal Testing Machine (UTM, TO-101, Siheung, Korea) with a 10 kgf/mm^2^ and crosshead of 1 mm/min. The BC and collagen membrane specimens were cut into 5 mm width × 20 mm length sizes. The specimens were tested in both dry and wet states after being immersed in distilled water for 48 h. Tensile strength measurement of membranes was analyzed by calculating the average maximum tensile strength of each sample (n = 5).

### 4.4. Experiment Animals

Forty male Sprague-Dawley rats (9 weeks old and body weight of 250–300 g) were used for the experiment. Prior to surgery, the experimental animals were randomly divided into four groups (n = 10/group). After surgery, five animals from each group had a healing period of 2 weeks, and the remaining five animals had a healing period of 8 weeks. The rats were acclimated in their individual plastic cage under laboratory conditions for 1 week before the experiment. This study followed the guidelines from the Pusan National University Institutional Animal Care and Use Committee (PNUIACUC-2015-0919).

### 4.5. Surgical Procedures

**Figure 8:**
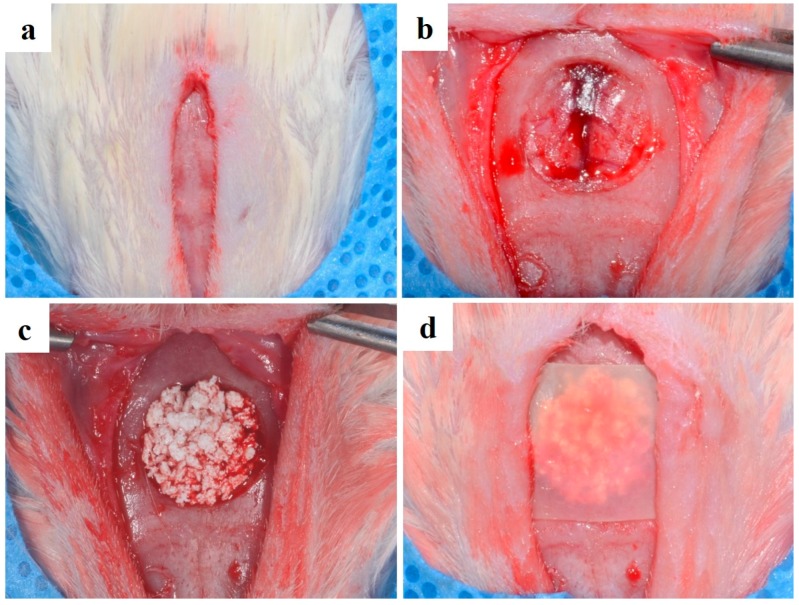
Operative procedure (**a**) full-thickness flap; (**b**) calvarial defect preparation using the 8 mm trephine bur; (**c**) bone graft material placement; (**d**) BC membrane placement.

All surgical procedures were performed under general anesthesia, which involved intramuscular injection of xylazine (Rumpun, Bayer Korea, Korea) and tiletamin-zolazepam (Zoletil, Vibac Laboratories, Carros, France). The rats were disinfected with betadine at the cranium site of surgery, and 2% lidocaine HCL (Yu-Han Co., Gunpo, Korea) containing 1:100,000 epinephrine was administered. The surgical site was incised, and a full-thickness flap of skin was reflected. The aneurysmal defect was formed in the central part of the cranium by using 8 mm trephine bur (3i Implant Innovation, Palm Beach Garden, FL, USA) under the injection. The defect sites were filled with 0.12 mg hydroxyapatite (HA)/Tricalcium phosphate (TCP) bone graft material (Bio-C, Cowellmedi Co., Ltd., Busan, Korea) and then covered with 10 × 10 mm collagen membrane or bacterial cellulose membrane (Figure 8). Twenty rats were sacrificed after 2 weeks of healing, and the rest were sacrificed after 8 weeks of healing.

### 4.6. Histomorphometric Analysis

After the animal were sacrificed, block sections of calvarial bone containing the membrane and surrounding tissues were dissected free and fixed in 10% neutral-buffered formalin. The specimens were demineralized with rapid acid clearing reagent and 14% EDTA, embedded in paraffin, and sectioned to a thickness of 5 μm. The central portion of each block was sectioned and stained with hematoxylin and eosin, respectively. Histologic slides were observed with an optical microscope (BX51, Olympus, Tokyo, Japan) and digitally captured with a CCD camera (SPOT Insight 2Mp, DIAGNOSTIC Instruments Inc., Sterling Heights, MI, USA). The captured images were analyzed using an image analysis program (IMT i-Solution, Inc., Vancouver, BC, Canada). For histometric analyses, ×20, ×40, and ×100 magnifications were used. The histometric analysis was conducted by the same professionally trained and blinded investigator. New bone area percentage (%) was analyzed and recorded within the area of defect (the area occupied by the new bones/the area of defect × 100 (%)).

### 4.7. Statistical Analysis

The null hypothesis set for this study was “There will be no difference in the bone regeneration ability according to the thickness of BC membrane, and the BC membrane will not be different even when comparing the bone regeneration ability with the existing collagen membrane”. The experimental data are presented as mean ± standard deviation. To examine the significances of thickness and tensile strength, Student’s *t*-test was applied (*p* < 0.05). In the in vivo study, the statistical software R (version 3.1.3) was used for statistical analysis. Nonparametric analysis introduced by Brunner and Langer was used for the group difference tests [38]. The analysis was conducted at a 5% significance level.

## 5. Conclusions

According to the histomorphometric analysis and mechanical experiment, the 0.10 mm bacterial cellulose membrane group manifested the highest level of new bone volume (mm³) and new bone area percentage (%). There was no significant difference among the other groups. Within the limitations of this study, these results may be used as a reference for determining efficient thickness of bacterial cellulose barrier membrane in guided bone regeneration procedure.

## Figures and Tables

**Table 1:** Mean (±SD) of neo-tissue (NT)/new-bone (NB) area (%) of groups at 2 and 8 weeks after surgery.

Variable	Group (n)	Mean ± SD	Median	*p*-Value
2 weeks **	Collagen (5)	1.54 ± 0.52	1.53	0.003
	0.10 mm (5)	3.45 ± 1.60	3.00	
	0.15 mm (5)	2.10 ± 0.25	2.06	
	0.20 mm (5)	1.62 ± 0.77	1.25	
8 weeks ***	Collagen (5)	9.08 ± 1.59	8.91	<0.001
	0.10 mm (5)	20.16 ± 3.41	19.41	
	0.15 mm (5)	10.70 ± 3.75	10.08	
	0.20 mm (5)	7.33 ± 2.63	7.12	

*** *p* < 0.001, ** *p* < 0.01, * *p* < 0.05.
